# Potassium fertilization modulates potato (*Solanum tuberosum* L. V7) yield and rhizosphere microbiome dynamics

**DOI:** 10.3389/fpls.2025.1618600

**Published:** 2025-06-26

**Authors:** Jing Yang, Xiaodong Han, Qi Li, Dong Wang, Yuankai Li, Ziyi Zhang

**Affiliations:** ^1^ College of Life Sciences, Inner Mongolia Agriculture University, Hohhot, Inner Mongolia, China; ^2^ Department of Food Engineering and Technology, Vocational and Technical College of Inner Mongolia Agricultural University, Hohhot, Inner Mongolia, China

**Keywords:** potassium fertilization, rhizosphere microbiome, potato yield, microbial diversity, sustainable agriculture

## Abstract

**Introduction:**

Potassium (K) is a critical macronutrient essential for enzymatic activation, photosynthesis, metabolite transport, and stress resistance in plants. While K is known to influence soil microbial communities, the mechanistic relationships between K fertilization regimes, rhizosphere microbiome assembly, and crop productivity remain to be elucidated. This study investigated the effects of varying K fertilization rates on potato yield and associated rhizosphere microbial community dynamics throughout the key developmental stages.

**Methods:**

A field experiment using potato (*Solanum tuberosum* L. V7) was conducted in Inner Mongolia, China, during the 2024 growing season. Five K fertilization treatments (0, 120, 180, 240, and 300 kg/ha K_2_O) were implemented using a randomized complete block design with three replicates. Rhizosphere soil samples were collected at seedling, tuber initiation, and tuber bulking stages. High-throughput sequencing of bacterial 16S rRNA and fungal ITS1 regions was performed to characterize microbial communities. Taxonomic composition, α-diversity, β-diversity, and linear discriminant analysis effect size was conducted to assess the correlation of potato yield and microbial diversity.

**Results:**

Potato yield exhibited a quadratic relationship with K application rate, reaching maximum productivity (66,786 kg/ha) at 240 kg/ha K_2_O. Bacterial communities, dominated by *Proteobacteria, Acidobacteriota, Actinobacteria*, and *Gemmatimonadota*, demonstrated notable resilience across treatments. Conversely, fungal communities displayed heightened sensitivity to K fertilization, with Shannon diversity indices negatively correlated with yield (r=-0.82, p<0.05). Moderate K application (180-240 kg/ha) significantly enhanced beneficial bacterial populations, particularly *Pseudomonas* species, while simultaneously suppressing pathogenic *Fusarium* and maintaining beneficial *Mortierellomycota*. Both bacterial and fungal communities exhibited distinct successional trajectories, with tuber expansion stage emerging as a critical transition point in community assembly.

**Discussion:**

This investigation establishes 180-240 kg/ha K_2_O as the optimal application rate for maximizing potato yield while maintaining balanced rhizosphere microbial communities. K influences microbial community structure through multiple mechanisms, including ion-hormone interactions, nutrient activation processes, and pathogen regulation. These findings provide a theoretical framework for developing precision K fertilization strategies that enhance agricultural productivity while promoting the stability of the rhizosphere microbiome in potato cultivation systems.

## 1 Introduction

Soil nutrient management is a cornerstone of modern agriculture, playing a pivotal role in enhancing crop yield and quality (Jing and Guillaume, 2021). Among essential macronutrients, potassium (K) is vital for plant growth and development. It facilitates enzyme activation (Mirza et al., 2020), photosynthesis (Xinxiang et al., 2020), and the synthesis and transport of metabolites within plants (Riya et al., 2022). These functions are critical for determining crop yield and quality, particularly in high-value crops, earning potassium the titles of “quality element” and “stress resistance element” (Jianglin et al., 2020). Studies have demonstrated that potassium fertilizer positively regulates lignin biosynthesis by modulating enzyme activity and gene expression, thereby enhancing stem strength and lodging resistance (Li X. et al., 2024). For instance, the foliar application of potassium nanoparticles significantly improved onion yield and quality by increasing the levels of nitrogen, phosphorus, potassium, proteins, carbohydrates, phenolics, pigments, flavonoids, and anthocyanins in onion bulbs (Salama et al., 2024). Similarly, potassium fertilization has been shown to boost the yield and quality of tea by promoting malic acid metabolism in the tricarboxylic acid cycle and enhancing the accumulation of carbohydrates and catechins in tea buds (Kailing et al., 2022).

In addition to its direct effects on plant growth, potassium also influences soil microbial communities and ecosystem functions. Potassium ions affect soil carbon cycling enzyme activities and microbial electron transport processes during reproduction, thereby impacting the decomposition of organic matter (Xi et al., 2023). Research comparing potassium organomineral fertilizers (OMF) with traditional potassium chloride (KCl) in tropical soils has revealed that OMF enhanced rhizosphere microbial diversity by altering soil nutrient availability and pH (Figueiredo Oliveira et al., 2025). Furthermore, the application of potassium fulvate significantly improved the relative abundance of beneficial microbial taxa such as *Ascomycota*, *Bacillaceae*, and *Proteobacteria* in continuous cropping systems (Jiao et al., 2024). Studies on wheat-maize rotations along a potassium gradient demonstrated that optimal potassium levels enhanced nutrient cycling processes mediated by specific bacterial taxa, including *Lactobacillus* during wheat seasons and *Nitrospira* during maize seasons. These findings underscore the potential of potassium fertilization not only to improve crop yield but also to reshape the structure and function of rhizosphere microbial communities (Li Z. et al., 2024).

Rhizosphere microorganisms play a crucial role as mediators between plants and soil, directly influencing nutrient uptake efficiency and plant health. Specific microbial communities can provide essential ecological support to plants through nutrient cycling and metabolic activities. For instance, nitrogen-fixing bacteria convert atmospheric nitrogen into bioavailable forms through nitrogenase systems, reducing dependency on chemical nitrogen fertilizers (Timofeeva et al., 2023; Chen et al., 2024). Phosphate-solubilizing microorganisms (PSM) release organic acids and enzymes to transform insoluble phosphorus into plant-accessible forms (Zhu et al., 2024), while potassium-solubilizing bacteria facilitate the release of potassium from soil minerals to enhance plant stress tolerance and productivity (Marzouk et al., 2025). Additionally, plant growth-promoting rhizobacteria synthesize phytohormones, including indole-3-acetic acid (IAA), gibberellins, and cytokinins to promote root elongation and reduce stomatal density (Mengyuan et al., 2023). Similarly, plant growth-promoting fungi, such as *Trichoderma*, stimulate auxin transport pathways to enhance photosynthesis, metabolism, and defense responses (Sheridan et al., 2022). However, the dynamic changes in rhizosphere microbial communities under varying fertilization regimes, and the correlation of these changes with crop productivity, remain insufficiently understood.

Potato (*Solanum tuberosum* L.) is a globally significant food and economic crop with high potassium requirements for optimal growth. Insufficient potassium fertilization can lead to rapid depletion of soil potassium reserves, particularly in resource-constrained agricultural systems, resulting in degraded soil physical properties and reduced crop productivity (Soumare et al., 2023). Potassium deficiency also weakens drought resistance in crops, further exacerbating agricultural challenges (Zhaoming et al., 2022). Conversely, excessive potassium application can cause environmental pollution, alter soil physicochemical properties (e.g., pH), reduce organic matter content, disrupt rhizosphere microbial functions critical for nutrient uptake, and increase susceptibility to pathogens like *Verticillium dahliae (* Ju et al., 2022; Das et al., 2023). For example, long-term fertilization studies have demonstrated that available potassium concentrations influence bacterial community diversity in sweet potato rhizosphere soils (Jing et al., 2023). Therefore, understanding the effects of varying levels of potassium fertilization on potato rhizosphere microbial communities is crucial for developing precision fertilization strategies that enhance resource use efficiency while promoting sustainable agricultural practices.

Previous studies have demonstrated that potato yield exhibits a quadratic response to potassium (K) fertilization, peaking at moderate application rates and declining with excess (Cao and Tibbitts, 1991; Zorb et al., 2014; Coulibali et al., 2020; Liu et al., 2023). Building on this established relationship, we investigated how rhizosphere microbial communities respond to varying K levels. We predicted that bacterial communities would remain more stable across potassium gradients than fungal communities, which were expected to be more sensitive to K induced changes. We also anticipated that microbial community dynamics would shift over time during potato development, particularly during the tuber initiation and bulking phases marking critical transitions in potassium mediated microbiome restructuring.

This study employed field experiments in conjunction with high-throughput sequencing to systematically analyze the diversity, composition, and functional characteristics of bacterial and fungal communities in potato rhizospheres subjected to varying levels of potassium fertilization. By integrating microbial data with yield measurements, this research aims to elucidate the interactions among potassium fertilization, rhizosphere microbiota, and potato productivity. The findings offer theoretical insights for optimizing fertilization practices to promote sustainable agriculture.

## 2 Materials and methods

### 2.1 Experimental site characterization

The field trial was conducted at the First Industrial Park Experimental Station (41°43’40”N, 111°36’6”E, and elevation: 1,417.2 m) of Xinyu Seed Industry Co., Ltd. in Wulanhua Town, Siziwang Banner, Inner Mongolia Autonomous Region. This region is characterized by a mid-temperate continental monsoon climate with an annual precipitation of 313.8 mm and 3,084 to 3,286 hours of sunshine. The sandy loam soil exhibited deep profiles and a loose texture, rendering it ideal for potato cultivation. During the 2024 growing season, the average temperature was recorded at 14°C, with wind speeds ranging from 3 to 4 on the Beaufort scale.

### 2.2 Experimental design

The field experiment was conducted using a ridge cultivation system with drip irrigation, without plastic mulch, at a planting density of 69,444 plants per hectare. Each plot measured 27 m² (6 m ridge length × 5 ridges), with ridge spacing of 90 cm and plant spacing of 16 cm, resulting in a total experimental area of 405 m². Five potassium fertilizer treatments were applied using K_2_SO^4^ (52% K_2_O) at rates of 0 (CK), 120 (T120), 180 (T180), 240 (T240), and 300 kg ha^-1^ (T300), while nitrogen (urea, 46% N) and phosphorus (diammonium phosphate, 18% N and 46% P_2_O_5_) were uniformly applied at rates of 250 kg ha^-1^ and 200 kg ha^-1^, respectively (**Table 1**). The potato cultivar ‘V7’ was planted using mechanical sowing and single-time manual fertilization. The experiment was arranged in a randomized complete block design with three replicates for each treatment.

**Table 1:** Gradient design of potassium fertilizer under different treatments.

Treatment	N(kg·ha^-1^)	P_2_O_5_(kg·ha^-1^)	K_2_SO^4^ (52%K_2_O) (kg·ha^-1^)
CK	250	200	0
T120	250	200	120
T180	250	200	180
T240	250	200	240
T300	250	200	300

CK, no potash fertilizer application; T120, fertilizer application rate of 120 kg·ha^-1^ ; T180, fertilizer application rate of 180 kg·ha^-1^; T240, fertilizer application rate of 240 kg·ha^-1^; T300, fertilizer application rate of 300 kg·ha^-1^.

### 2.3 Rhizosphere soil collection

Rhizosphere soil samples were collected at seedling (June 22, 2024), tuber initiation (July 12, 2024), and tuber bulking stage (August 10, 2024) of potato development. For each treatment, three potatoes with similar growth vigor were excavated, and rhizosphere soil was brushed off, homogenized, and divided into three subsamples. The rhizosphere soil of potatoes (attached to the root surface) was collected by the shaking-off method (Morgan et al., 2018). It was then stored in sterile bags labeled with treatment codes and transported on dry ice. Finally, it was frozen and preserved at -80°C for soil DNA extraction and metabolite analysis (Cindy et al., 2012).

### 2.4 Amplification and sequencing

Genomic DNA was extracted from 500 mg of rhizosphere soil using the E.Z.N.A.^®^ Soil DNA Kit (Omega Biotek, D5625-01). DNA quality and concentration were assessed via agarose gel electrophoresis and a OneDrop^®^ spectrophotometer. The bacterial 16S rRNA V4 region was amplified using primers 341F (5’-CCTACGGGNGGCWGCAG-3’) and 806R (5’-GGACTACHVGGGTWTCTAAT-3’), while the fungal ITS1 region was amplified with primers ITS1F (5’-CTTGGTCATTTAGAGGAAGTAA-3’) and ITS1R (5’-GCTGCGTTCTTCATCGATGC-3’). Amplicons (400-450 bp for bacteria, 310 bp for fungi) were purified via agarose gel electrophoresis.

### 2.5 Potato yield determination

Potatoes were harvested at physiological maturity on September 28, 2024, when more than 80% of the foliage had naturally senesced. For yield assessment, three representative potato plants exhibiting uniform growth characteristics were randomly selected from each plot central rows to minimize edge effects, yielding nine sample plants per treatment across three replicate plots. Tubers were carefully hand-excavated to prevent mechanical damage, with adhering soil gently removed using soft brushes to maintain tuber integrity. The plot yield was calculated by multiplying the average per-plant yield by the established planting density.

### 2.6 Statistical analysis

Sequencing was performed on the Illumina NovaSeq 6000 platform (2 × 250 bp PE) by Jisi Huiyuan Biotechnology Co., Ltd. (Nanjing). Raw reads were merged, quality-filtered, and clustered into Amplicon Sequence Variants (ASVs) using QIIME2 with the DADA2 pipeline. Taxonomic annotation was conducted against the SILVA 138 (bacteria) and UNITE v9.0 (fungi) databases, with unclassified ASVs excluded from downstream analyses.

Microbial diversity analyses were conducted at uniform sequencing depth (rarefied to 35,000 sequences/sample for bacteria and 25,000 for fungi). For α-diversity (Li et al., 2013) (Chao1 richness, Shannon index), statistical significance between potassium treatments and growth stages was assessed via two-way ANOVA with Tukey’s HSD *post-hoc* tests (p<0.05), implemented in R v4.3.2. β-diversity analysis utilized Bray-Curtis dissimilarity matrices subjected to Principal Coordinates Analysis (PCoA) (Minchin, 1987), with treatment effects quantified through PERMANOVA using the Adonis function (vegan package, R) with 999 permutations and Bonferroni-corrected pairwise comparisons. Linear Discriminant Analysis Effect Size (LEfSe) identified taxa differentially abundant between treatments (LDA score >4.0, Kruskal-Wallis p<0.05).

Polynomial regression analysis between yield (Y) and potassium fertilizer application (X) was implemented in Python, with the model: Y = β_0_ + β_1_ X + β_2_ X^2^ + ∈ (Jianqin et al., 2024). Model parameters were estimated via ordinary least squares using numpy.polyfit, with goodness-of-fit evaluated through coefficient of determination (R^2^) and model significance tested via ANOVA (F-test, scipy.stats.pearsonr). Correlation analyses employed a tiered approach: Pearson’s correlation (parametric) for normally distributed variables (validated via Shapiro-Wilk test, p>0.05) and Spearman’s rank correlation (non-parametric) otherwise. All correlation matrices incorporated Benjamini-Hochberg false discovery rate (FDR) correction (q<0.1) to account for multiple comparisons. Microbial-yield relationships were further visualized through SparCC co-occurrence networks (∣*ρ*∣>0.6, p<0.01).

## 3 Results

### 3.1 Overall analysis of 16S rDNA and ITS

High-throughput sequencing of bacterial 16S rDNA (V4 region) and fungal ITS1 regions across 15 rhizosphere soil samples generated robust datasets for microbial community analysis. Bacterial sequencing with 341F-806R primers produced 2,110,432 raw reads, yielding 1,779,902 high-quality non-chimeric ASVs after filtering and denoising, with an average of 118,660 ASVs per replicate (**Supplementary Table S1**). Fungal sequencing with the ITS1F-ITS1R primers generated 2,265,804 raw reads, resulting in 2,166,757 non-chimeric ASVs, averaging 144,450 ASVs per replicate (**Supplementary Table S2**). These results provide reliable datasets for subsequent analyses of diversity and community structure.

### 3.2 Alpha diversity analysis

The alpha diversity of potato rhizosphere microbial communities under varying potassium fertilization treatments was evaluated across seeding, tuber initiation, and tuber expansion stages (**Table 2**). High Goods-Coverage values (>0.995 for bacteria and >0.999 for fungi) confirmed adequate sequencing depth across all treatments and growth stages. For bacterial communities, richness indices (Chao1 and Ace) were highest in CK and T300 treatments at the seeding stage. During the tuber initiation stage, bacterial richness decreased slightly across treatments, though T300 maintained the highest values. At the tuber expansion stage, bacterial richness increased significantly, with T180 and T240 treatments showing nearly a twofold enhancement compared to the other two stages. Shannon indices remained relatively stable across all treatments and growth stages, indicating consistent bacterial community diversity.

**Table 2:** Alpha diversity analysis of potato inter-root microorganisms under different fertilization treatments at different growth periods.

Strains	Treatment	Seedling stage			Tuberogenesise			Tuber expansion stage		
		Chao 1	Ace	Goods-Coverage	Chao 1	Ace	Goods-Coverage	Chao 1	Ace	Goods-Coverage
Bacteria	CK	5742.92Aa	5763.31Aa	0.9986Ab	4564.68Ab	4626.61Aab	0.9956Aa	8176.44Aab	8267.37 Aab	0.9967 Aab
	T120	5169.79Aa	5176.39Aa	0.9996Aa	4095.27Ab	4130.81Ab	0.9973Aa	7657.60 Ab	7737.65Ab	0.9968 Aab
	T180	5190.36Aa	5214.97Aa	0.9985Ab	4582.2Ab	4646.55Aab	0.9954Aa	8903.60 Aa	9031.25 Aa	0.9953Ab
	T240	5280.84Aa	5299.92Aa	0.9988Aab	4391.21Ab	4432.62Ab	0.9971Aa	8999.52 Aa	9131.30 Aa	0.9951Ab
	T300	5685.05Aa	5709.41Aa	0.9985Ab	5584.78Aa	5607.18Aa	0.9979Aa	8016.15Aab	8070.92 Aab	0.9980Aa
Fungi	CK	709.37Aa	709.59Aa	0.9999Aa	747.66Aa	746.12 Aa	0.9996Aab	809.07 Aa	809.90 Aa	0.9999Aa
	T120	574.82Aa	575.32Aa	0.9999Aab	730.01Aa	730.20 Aa	0.9999Aab	643.10 Aa	644.17 Aa	0.9999 Aab
	T180	668.29Aa	668.19Aa	0.9999Abc	713.23Aa	713.38 Aa	0.9999 Aa	740.22 Aa	740.92 Aa	1.0000Aa
	T240	637.93Aa	638.96Aa	0.9998Ac	727.40Aa	730.01 Aa	0.9996 Ab	829.94 Aa	833.03 Aa	0.9998Ab
	T300	879.32Aa	878.87Aa	0.9999Aabc	409.75Bb	411.44 Bb	0.9998Aab	505.56 Aa	506.45 Aa	0.9999 Aab

Unit, piece; values are the mean of three replicates. CK, no potash fertilizer application; T120, fertilizer application rate of 120 kg/ha ; T180, fertilizer application rate of 180 kg/ha; T240, fertilizer application rate of 240 kg/ha; T300, fertilizer application rate of 300 kg/ha. Values are reported as repeated mean. According to Spass of Duncan’s test, the average of the different letters (such as a, b, and c) in each column was significantly different at P<0.05;the average of the different letters (such as A, B, and C) in each column was highly significantly different at P<0.

Fungal communities exhibited distinct trends compared to bacteria. At the seeding stage, fungal richness was highest in T300, while T120 showed lower diversity. During the tuber initiation stage, fungal richness in T300 declined sharply, whereas other treatments displayed similar levels of richness and diversity. By the tuber expansion stage, fungal richness increased in several treatments, with T240 demonstrating the highest values. However, fungal Shannon indices were consistently lower than those of bacteria throughout all growth stages. These findings suggest that moderate potassium application (T180 and T240) enhances bacterial richness during critical growth periods while excessive potassium application (T300) negatively impacts fungal diversity.

### 3.3 Beta Diversity of microbial community in rhizosphere soils

**Figure 1:**
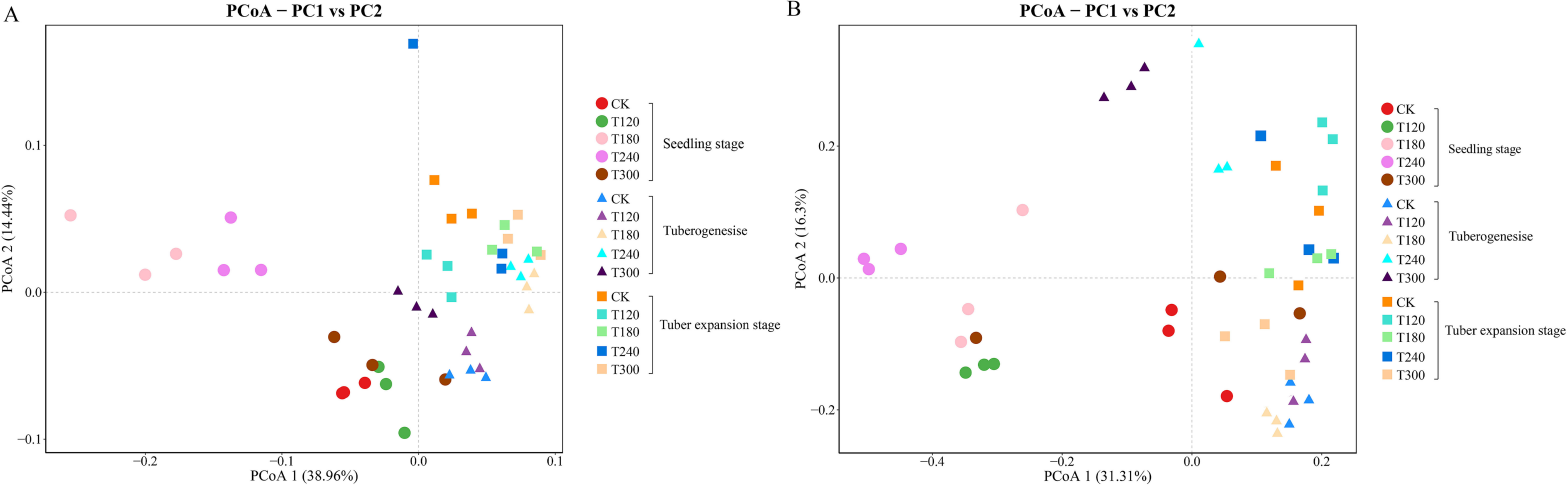
Principal coordinates analysis (PCoA) of rhizosphere microbial communities. PCoA plots based on Bray-Curtis distances showing **(A)** bacterial communities and **(B)** fungal communities at the genus level in potato rhizosphere. Colors represent potassium fertilizer treatments. Symbols indicate growth stages: circles= seedling, triangles= tuberogenesis, squares= tuber expansion.

Beta diversity was analyzed using Principal Coordinate Analysis (PCoA) to assess the bacterial and fungal communities in potato rhizosphere soils. The PCoA results highlighted significant effects of potassium fertilization across different growth stages. At the genus level, PCoA1 accounted for 38.96% of the variance and PCoA2 accounted for 21.44% in bacterial community composition (**Figure 1A**), whereas PCoA1 explained 31.33% and PCoA2 explained 16.30% of the variance in fungal community composition (**Figure 1B**).

During the seedling stage, microbial communities were relatively dispersed across different potassium treatments, suggesting a significant early influence of potassium on rhizosphere community structure. As potatoes progressed through tuber formation and enlargement stages, microbial communities showed convergence trends, indicating that plant developmental stages exerted stronger selective pressures on rhizosphere microbiomes, partially offsetting initial differences caused by potassium treatments. Notably, the control group (CK) maintained relatively stable positioning across all growth stages, while treatments T120, T180, T240, and T300 showed distinct shifts between early and later growth phases, demonstrating long-term effects of potassium fertilization on microbial community structure. This pattern was consistent across taxonomic levels and biological groups, supporting the conclusion that potassium serves as a key environmental factor systematically influencing rhizosphere microbial community succession.

### 3.4 Alterations in bacterial and fungal community composition

**Figure 2:**
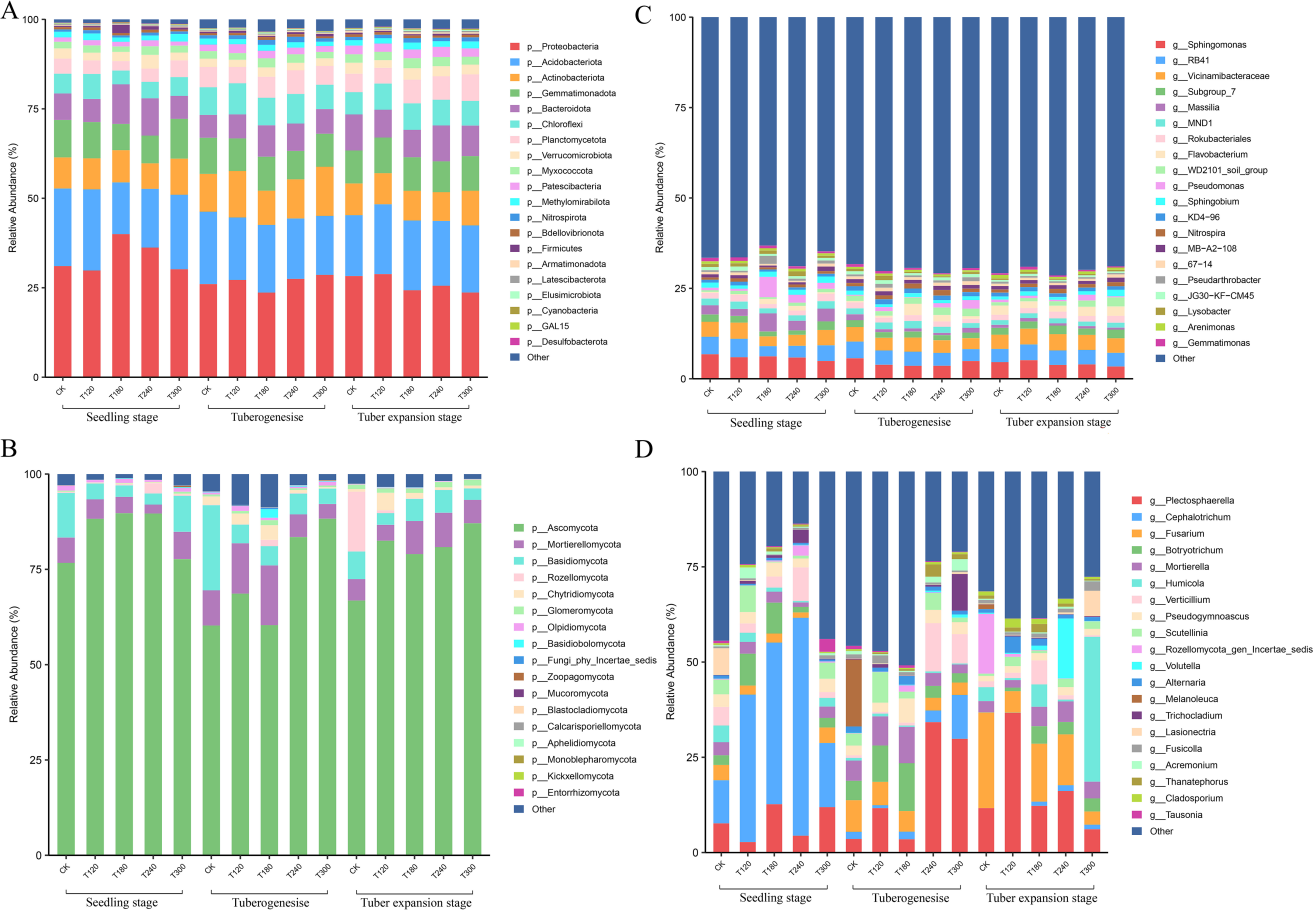
Rhizosphere microbial community composition of potatoes at different growth stages under varying potassium fertilizer treatments. Relative abundance (%) of bacterial communities at **(A)** phylum and **(B)** genus levels, and fungal communities at **(C)** phylum and **(D)** genus levels in potato rhizosphere soil. Communities were analyzed across three developmental phases (seedling, tuberogenesis, and tuber expansion stages) and five potassium fertilizer treatments (CK, T120, T180, T260, and T320). Bar charts display the top 20 most abundant taxa. The y-axis shows relative abundance percentages.

The taxonomic composition of bacterial and fungal communities in potato rhizosphere soil showed distinct dynamics across growth stages under varying potassium fertilizer treatments. At the bacterial phylum level, the dominant groups identified were Proteobacteria, Acidobacteriota, Actinobacteria, and Gemmatimonadota. Proteobacteria dominated T180 and T240 treatments at the seedling stage but declined progressively through tuber formation and expansion phases, while Planctomycetota increased notably during tuber expansion (**Figure 2A**). At the genus level, *Sphingomonas*, *RB41*, *Vicinamibacteraceae*, and *Subgroup_7* were the dominant genera. Key genera including Subgroup_7 and Flavobacterium surged during tuber expansion, whereas Sphingomonas and Massilia exhibited marked declines, particularly pronounced in T180 and T240 treatments (**Figure 2B**).

Fungal communities were dominated by Proteobacteria, Acidobacteriota, and Actinobacteriota phyla. During the potato growth stages, Proteobacteria decreased gradually, while Planctomycetota, Verrucomicrobiota, and Myxococcota increased (**Figure 2C**). Fungal communities exhibited greater compositional shifts and were dominated by the Ascomycota and Basidiomycota phyla. Cephalotrichum prevalence at the seedling stage (especially in T120-T240 treatments) gave way to Fusarium and Plectosphaerella dominance during tuber expansion (**Figure 2D**). These changes were most pronounced in T180 and T240 treatments, showing strong potassium-dependent patterns.

In general, bacterial communities underwent gradual compositional adjustments, while fungal populations displayed abrupt, treatment-specific restructuring during critical growth phases. The tuber expansion stage emerged as a pivotal transition point for both microbial kingdoms, with potassium fertilization intensity (particularly T180-T240 ranges) driving measurable abundance fluctuations across taxonomic groups.

### 3.5 LEfSe analysis of bacterial and fungal communities

**Figure 3:**
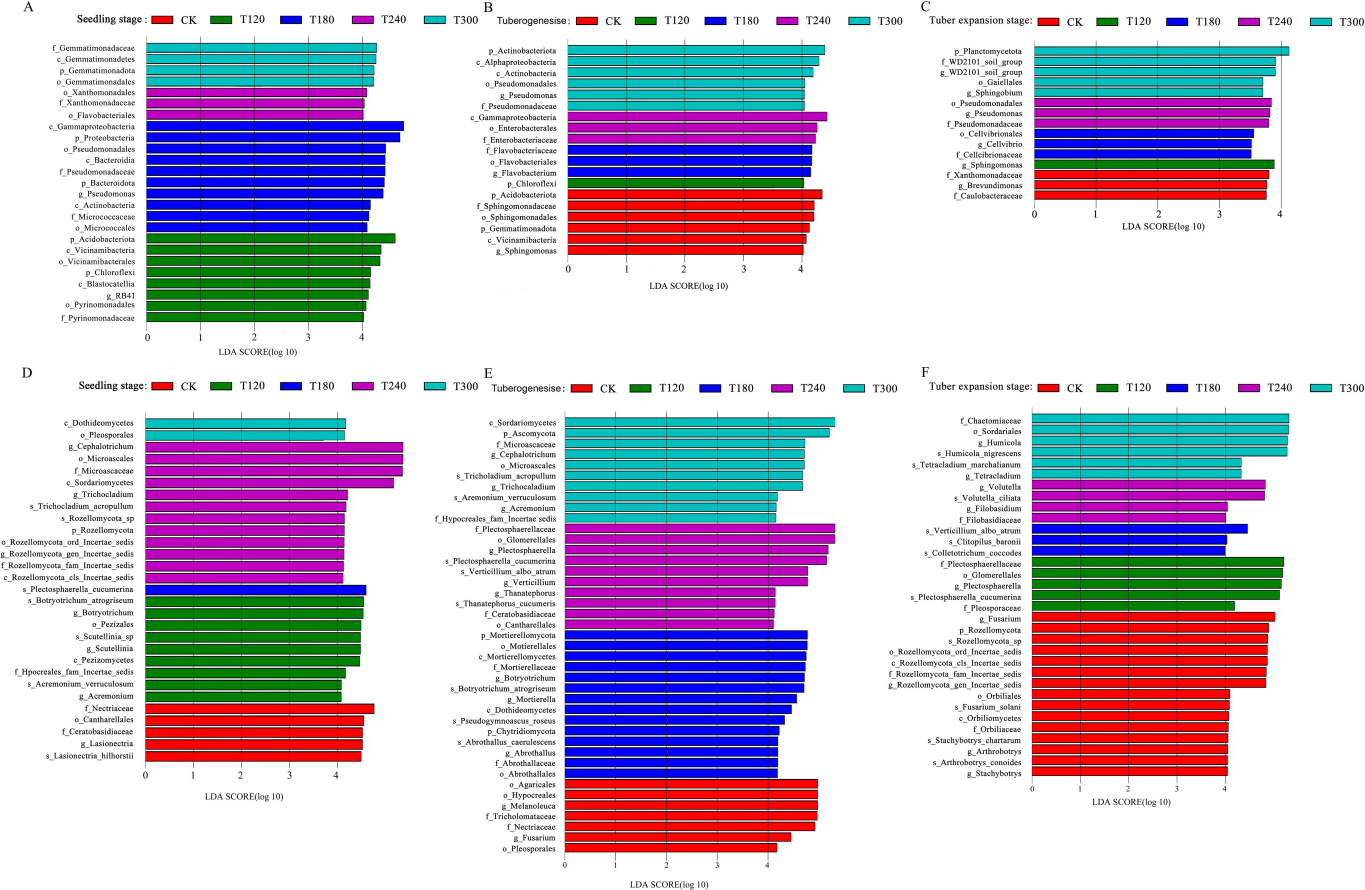
Linear discriminant analysis effect size (LEfSe) of microbial communities in potato root-zone soil under different potassium treatments (CK, T120, T180, T240, T300) across three growth stages. Panels **(A–C)** show differential abundance of rhizobacterial communities (LDA score >4) during seedling stage **(A)**, tuberogenesis **(B)**, and tuber expansion stage **(C)**. Panels **(D–F)** display differential abundance of rhizofungal communities during seedling stage **(D)**, tuberogenesis **(E)**, and tuber expansion stage **(F)**. Bar colors represent the specific potassium treatment where each taxon was significantly enriched, while bar length indicates the magnitude of differential abundance between treatments.

The Linear Discriminant Analysis Effect Size (LEfSe) method was employed to investigate microbial community differences in the potato rhizosphere soil, identifying significantly enriched taxa and their relative abundances. During the seedling stage, LEfSe analysis revealed 25 distinct bacterial taxa with linear discriminant analysis (LDA) scores >4, including key groups such as *c_Gammaproteobacteria*, *p_Proteobacteria*, and *p_Acidobacteriota*. Notably, treatments T180 and T240 enriched 13 bacterial taxa, suggesting that these treatments enhanced bacterial diversity by modifying the soil microenvironment (**Figure 3A**). During the tuber formation stage, 19 enriched bacterial taxa with LDA >4 were identified. T300 and the control (CK) enriched 11 bacterial taxa, while T120, T180, and T240 collectively enriched only seven taxa (**Figure 3B**). By the tuber expansion stage, only one significantly enriched taxon (p_Planctomycetota) with LDA >4 enriched (**Figure 3C**). Interestingly, across all growth stages, *Pseudomonas* was consistently enriched, particularly under T180 and T240 treatments. This is significant as *Pseudomonas* is well-known for its role in nutrient cycling and plant growth promotion.

The fungal community also exhibited distinct responses to K fertilization across potato growth stages. During the seedling stage, *g_Cephalotrichum*, *o_Microascales*, and *f_Microascaceae* were strongly associated with T240 treatments, while *s_Plectosphaerella cucumerina* was predominantly enriched under T180 (**Figure 3D**). As plants progressed to tuberogenesis, fungal diversity increased significantly, with a total of 41 fungal taxa enriched at LDA >4 (**Figure 3E**). By the tuber expansion stage, the fungal community became highly specialized. CK treatments distinctly enriched several fungal groups, including *Fusarium* and *Ophiostoma* (**Figure 3F**). These findings highlight that optimized K fertilization strategies can harness microbial community dynamics to enhance sustainable potato production.

### 3.6 Potato yield under different potassium treatments

**Figure 4:**
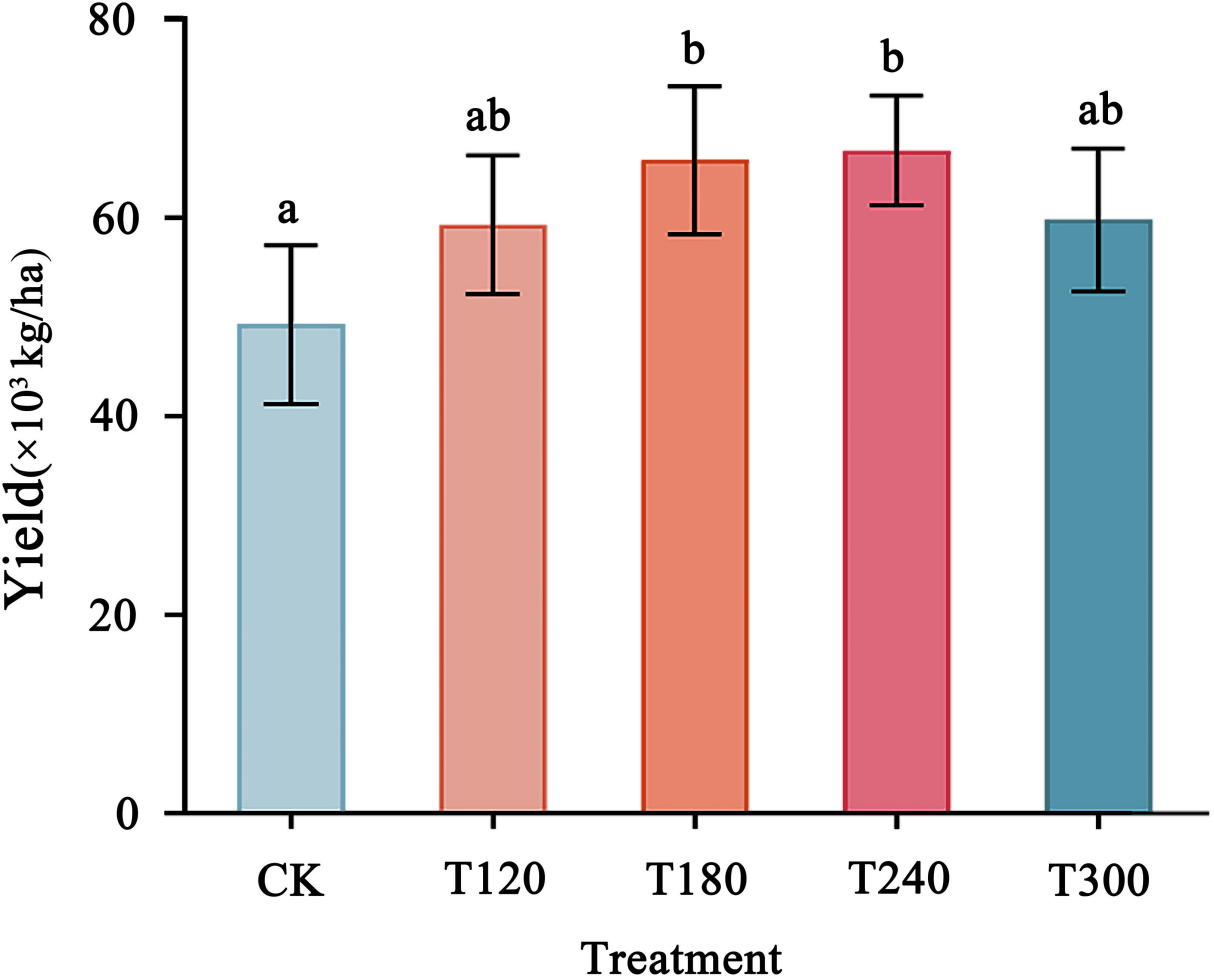
Potato yield in response to different potassium fertilizer application rates. Bars with different letters (a, b) indicate statistically significant differences (p<0.05). Error bars represent standard deviation.

Potato yield varied significantly across the K fertilization treatments, showing a trend of initial increase followed by a decline as potassium application rates increased (**Figure 4**). The highest yield was observed under the T240 treatment (66,786 kg/ha), which significantly outperformed the CK and other treatments. Similarly, the T180 treatment also resulted in a substantial yield increase compared to CK. However, excessive K application (T300) led to a decrease in yield, indicating that over-application may negatively impact potato productivity. These results highlight the importance of optimizing potassium fertilizer application rates, with moderate levels (T180–T240) being most effective in achieving maximum potato yield.

### 3.7 Correlation analysis of potato yield and microbial diversity

**Figure 5:**
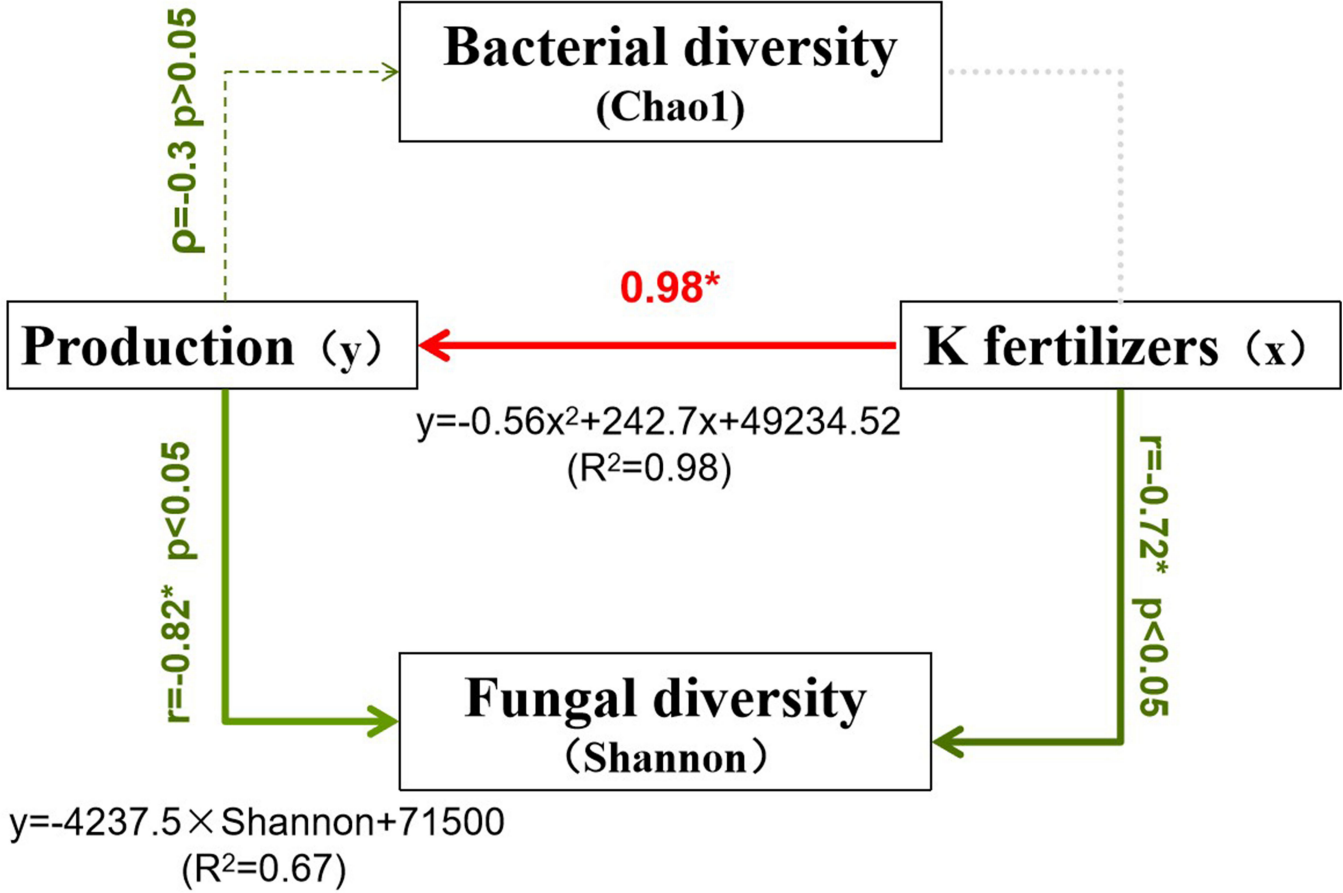
Structural equation model showing relationships between potato yield, soil microbial diversity, and potassium fertilizer application. K fertilizers strongly increase potato production (0.98*, R²=0.98) while negatively affecting fungal diversity (r=-0.72*, p<0.05).

The correlation analysis conducted using Python indicated that potato yield responds to potassium fertilizer (K_2_O) application in a quadratic manner, described by the equation: y = -0.56x² + 242.7x + 49,234.52 (R² = 0.98). This suggests that increasing K within a certain range significantly enhances yield, which peaks near the T240 treatment before declining at higher application rates, thereby highlighting the existence of an optimal application level. Furthermore, bacterial diversity showed a weak negative correlation with yield (Spearman’s ρ = -0.3, p > 0.05), suggesting no significant linear relationship, although moderate bacterial diversity was observed during the high-yield stages (T180–T240). In contrast, fungal diversity was strongly and negatively correlated with yield (correlation coefficient = -0.82), implying that greater fungal diversity may be linked to lower yields, potentially due to shifts in pathogenic or functional fungal groups (**Figure 5**).

Further analysis demonstrated that K fertilizer had no significant effect on bacterial diversity (as measured by the Chao1 index), but it did significantly suppress fungal diversity (correlation coefficient = -0.72). Based on these findings, the recommended potassium application rate is 180–240 kg/ha K_2_O, which achieves the highest yields (65,748–66,786 kg/ha) while maintaining a relatively balanced microbial community. Notably, bacterial diversity was highest under low potassium (CK) conditions, while fungal diversity’s strong negative association with yield warrants further investigation into the roles of specific fungal groups.

## 4 Discussion

This study provides critical insights into the complex interactions between potassium fertilization, rhizosphere microbiome dynamics, and potato productivity, establishing a foundation for precision nutrient management in sustainable agricultural systems. Our findings reinforce the established quadratic relationship between potassium (K) application rates and potato yield by providing empirical validation and quantifying the response through a fitted quadratic model: Y = aK² + bK + c (R² = 0.98), where Y = yield and K = potassium rate, with optimal yields occurring at 180-240 kg/ha K_2_O. This discovery has immediate practical implications for potato growers worldwide, as it provides quantitative guidelines for maximizing productivity while minimizing input costs and environmental impact. Our results show that exceeding 240 kg/ha K_2_O leads to yield decline, likely due to ionic imbalances causing reactive oxygen species accumulation and reduced antioxidant enzyme activity (Skrobacz et al., 2024). This finding addresses a critical knowledge gap in potato nutrition management, where previous studies lacked comprehensive integration of yield responses with rhizosphere microbiome dynamics.

The bacterial communities exhibited remarkable stability across different K treatments, with no significant correlation between Chao1 diversity index and K application rates (ρ = -0.3, p > 0.05), suggesting functional redundancy or resilience within bacterial populations. Planctomycetota and Proteobacteria dominated across all growth stages, indicating their crucial role in nutrient cycling processes such as nitrogen fixation and organic matter decomposition, regardless of K input levels (Hu, 2025). Recent genomic research has revealed that soil bacteria maintain functional stability through horizontal gene transfer of potassium homeostasis-related genes (Jurdzinski et al., 2023). During the tuber formation stage, T180-T240 treatments significantly increased bacterial diversity, particularly beneficial *Pseudomonas* strains capable of potassium uptake and IAA production, while *Flavobacterium* became dominant during tuber expansion, producing potassium-activated amylases that directly promote starch accumulation in tubers (Vincze et al., 2024).

In contrast to bacteria, fungal communities showed greater sensitivity to K fertilization, with Shannon diversity index significantly negatively correlated with yield (r = -0.82, p < 0.05). The abundance of *Ascomycota* decreased with increasing K levels, which may be attributed to their limited number of potassium transporter gene copies making them more susceptible to K fluctuations. LEfSe analysis revealed significant differences in fungal community structure across treatments and growth stages, with T180 and T240 treatments establishing a beneficial balance by suppressing pathogenic *Fusarium* while maintaining beneficial *Mortierellomycota*. Potassium influences microbial communities and crop growth through multiple pathways, including ion-hormone interactions, microbial nutrient activation, pathogen regulation. These findings establish that 180-240 kg/ha of K fertilizer suppresses *Fusarium* while preserving beneficial *Mortierella* (Ozimek and Hanaka, 2020).

With potato being the fourth most important global food crop, our findings have far-reaching implications for food security. The precision fertilization strategies developed here could enhance global potato productivity while reducing environmental footprint, contributing to sustainable intensification goals. The ability to maintain beneficial microbial communities while maximizing yields addresses the urgent need for agricultural practices that support both human nutrition and ecosystem health.

This research enables the development of precise K management strategies tailored to stage-specific microbial dynamics, maintaining bacterial nutrient cycling functions, balancing pathogenic and beneficial fungal ratios, and ultimately achieving precision fertilization (Liu et al., 2021). Future research should incorporate metabolomics of root exudates (Möller et al., 2024) and spatial transcriptomics to further elucidate the spatiotemporal dynamics of potassium-mediated microbiome regulation (Ji et al., 2025).

## Data Availability

The datasets presented in this study can be found in online repositories. The names of the repository/repositories and accession number(s) can be found in the article/supplementary material.
