# Supplementary material for: Potassium fertilization modulates potato (Solanum tuberosum L. V7) yield and rhizosphere microbiome dynamics
Source: Front Plant Sci. 2025 Jun 26;16:1618600. doi: 10.3389/fpls.2025.1618600 (PMC12240928; doi:10.3389/fpls.2025.1618600)
Supplement: Supplementary file 1 [file Table1.doc]

**Potassium Fertilization Modulates potato (*Solanum tuberosum* L. V7) Yield and Rhizosphere Microbiome Dynamics**

| Periods | Treatment | input | filtered | denoised | non-chimeric |
| --- | --- | --- | --- | --- | --- |
| Seedling stage | CK | 173855 | 165325 | 155443 | 155189 |
|  | T120 | 150453 | 142668 | 133708 | 133633 |
|  | T180 | 177100 | 167712 | 157700 | 157309 |
|  | T240 | 171344 | 161713 | 152074 | 151850 |
|  | T300 | 171160 | 162789 | 152321 | 151853 |
| Tuberogenesise | CK | 87341 | 81146 | 68373 | 67943 |
|  | T120 | 82234 | 76464 | 65381 | 65044 |
|  | T180 | 79886 | 80441 | 67483 | 67071 |
|  | T240 | 83770 | 77337 | 64676 | 64281 |
|  | T300 | 87488 | 81393 | 64144 | 63645 |
| Tuber expansion stage | CK | 165634 | 159731 | 139164 | 136765 |
|  | T120 | 168516 | 162320 | 144457 | 142960 |
|  | T180 | 176073 | 169764 | 147650 | 145855 |
|  | T240 | 176653 | 169776 | 146753 | 144384 |
|  | T300 | 158925 | 153396 | 133658 | 132120 |
| SUM | | 2110432 | 2011975 | 1792985 | 1779902 |

**Table S1** Bacterial statistical data

Table S1. The first column period is potato growing period; The second column treatment is the sample name; The third column input is the average value of the raw data; The fourth column filtered is the amount of data after removing the low-quality sequence. The fifth column denoised the amount of sequence data after denoising, that is, the effective sequence amount; The sixthly column non-chimeric is the sequence quantity after the removal of chimera, that is the sequence quantity of high quality.

**Table S2** Fungi statistics data

| Periods | sample-id | input | filtered | denoised | non-chimeric |
| --- | --- | --- | --- | --- | --- |
| Seedling stage | CK | 166564 | 160519 | 159771 | 159241 |
|  | T120 | 183592 | 177971 | 177587 | 177580 |
|  | T180 | 215149 | 208935 | 208456 | 208381 |
|  | T240 | 200775 | 195268 | 194813 | 194788 |
|  | T300 | 198763 | 191679 | 190906 | 190653 |
| Tuberogenesise | CK | 142225 | 135417 | 134697 | 133997 |
|  | T120 | 105382 | 100369 | 99668 | 99619 |
|  | T180 | 86321 | 82307 | 81715 | 81713 |
|  | T240 | 113109 | 107630 | 107031 | 106345 |
|  | T300 | 118694 | 112864 | 112329 | 111701 |
| Tuber expansion stage | CK | 138630 | 134651 | 133661 | 131596 |
|  | T120 | 143885 | 139863 | 139137 | 138542 |
|  | T180 | 138805 | 134761 | 133982 | 132853 |
|  | T240 | 166658 | 161805 | 160946 | 159124 |
|  | T300 | 147252 | 142672 | 141992 | 140624 |
|  | SUM | 2265804 | 2186711 | 2176691 | 2166757 |

Table S2. The first column period is potato growing period; The second column treatment is the sample name; The third column input is the average value of the raw data; The fourth column filtered is the amount of data after removing the low-quality sequence. The fifth column denoised the amount of sequence data after denoising, that is, the effective sequence amount; The sixthly column non-chimeric is the sequence quantity after the removal of chimera, that is the sequence quantity of high quality.
